# Activating primary care COPD patients with multi-morbidity through tailored self-management support

**DOI:** 10.1038/s41533-020-0171-5

**Published:** 2020-04-03

**Authors:** Sameera Ansari, Hassan Hosseinzadeh, Sarah Dennis, Nicholas Zwar

**Affiliations:** 1 School of Public Health and Community Medicine, UNSW Sydney, Sydney, NSW Australia; 2 School of Health and Society, University of Wollongong Australia, Wollongong, NSW Australia; 3 Sydney School of Health Sciences, Faculty of Medicine and Health, The University of Sydney, Sydney, NSW Australia; 4 Ingham Institute for Applied Medical Research, Sydney, NSW Australia; 5 South Western Sydney Local Health District, Sydney, NSW Australia; 6 Centre for Primary Health Care and Equity, UNSW Sydney, Sydney, NSW Australia; 7 Faculty of Health Sciences and Medicine, Bond University, Gold Coast, QLD Australia

**Keywords:** Chronic obstructive pulmonary disease, Patient education, Health services, Translational research, Public health

## Abstract

Given the dearth of COPD self-management interventions that specifically acknowledge multi-morbidity in primary care, we aimed to activate COPD patients through personalised self-management support that recognised the implications of co-morbidities. This single-group experimental study included patients aged 40−84 with a spirometry diagnosis of COPD and at least one co-morbidity. A self-management education programme for COPD in the context of multi-morbidity, based on the Health Belief Model, was tailored and delivered to participants by general practice nurses in face-to-face sessions. At 6 months’ follow-up, there was significant improvement in patient activation (*p* < 0.001), COPD-related quality of life (*p* = 0.012), COPD knowledge (*p* < 0.001) and inhaler device technique (*p* = 0.001), with no significant change in perception of multi-morbidity (*p* = 0.822) or COPD-related multi-morbidity (0.084). The programme improved patients’ self-efficacy for their COPD as well as overall health behaviour. The findings form an empirical basis for further testing the programme in a large-scale randomised controlled trial.

## Introduction

Chronic obstructive pulmonary disease (COPD) is currently the third leading cause of mortality worldwide, with 3 million people having died of the disease in 2016^1^. COPD often occurs in the presence of co-morbidities, which might have concordant or discordant pathophysiology^2^. In Australia, COPD was the fifth leading cause of death in 2015^3^, with 91% of those reported to have COPD experiencing at least one co-morbidity^4^. Co-morbidities contribute to poor health status and higher healthcare utilisation^5^, and aggravate the debilitating nature of COPD^6^.

Since the majority of the Australian population visit their general practitioner (GP) more than once a year^7^, general practice offers the continuity of care needed for interventions aimed at enhancing patients’ self-management of chronic disease. With practice nurses (PNs) increasingly contributing to chronic disease management, nurse-delivered self-management support for coordinated care of patients with COPD could lead to significant uptake of interventions shown to improve health outcomes such as smoking cessation, pulmonary rehabilitation and influenza vaccination^8,9^. For such interventions to be effective, patients need to be involved in active discussion that would enhance their self-efficacy for management of COPD^10^.

A qualitative study by the authors found that people with early-stage COPD have difficulty recognising the importance of the condition and its long-term implications and COPD may be given low priority compared to their other co-morbidities^11^. While self-management interventions for patients with COPD can improve health-related quality of life and reduce respiratory-related hospital admissions^12^, most prior studies have focussed on COPD alone and only included patients with moderate or severe disease^13–16^. A meta-analysis of seven studies of community-based, self-management interventions among primary care COPD patients found no between-group difference in health-related quality of life at final follow-up^17^. There is evidence that a multi-faceted approach is needed to bring about change in health behaviour among those with COPD and providing them with only disease knowledge is not enough^18^. Due to the heterogeneous nature of the disease, effective self-management for COPD needs personalised education and active guidance^19^. Given that most patients with chronic respiratory disease have other chronic conditions^20^, it is essential to include strategies for coping with multi-morbidity in COPD self-management interventions^11,21^.

To date, no research study has provided a behaviour change theory-based, tailored, self-management support for COPD in the context of multi-morbidity, particularly in the primary care setting. The Activating Primary Care COPD Patients with Multi-morbidity (APCOM) study aimed to activate patients in regard to their COPD while acknowledging and tailoring support to recognise the implications existing of co-morbidities. It was hypothesised that at 6 months’ follow-up after a tailored, self-management education programme, participating patients would have: (i) better activation in terms of their COPD-related health behaviour, (ii) improved knowledge and self-management capacity of COPD, and (iii) increased self-efficacy in terms of their overall health behaviour.

## Results

### Recruitment

**Fig. 1:**
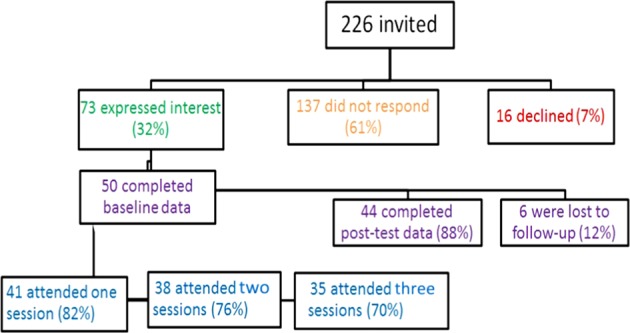
Recruitment of patient participants and uptake of the programme. It shows the response rate of patient participants and rate of completion of the APCOM programme.

Among 61 practices invited to participate, 13 (21%) consented to take part, 17 (28%) did not respond and 31 (51%) declined. One practice withdrew due to lack of potential patients after the PN conducted a medical record search. A total of 226 patients were invited to participate in the study (Fig. 1), of whom 50 eventually participated in the study. Each PN managed between two and seven patients during the study.

### Patient characteristics

Of 50 patients at baseline, 44 completed 6 months’ follow-up. Their baseline characteristics are summarised in Table 1. There were a total 90 co-morbidities among the 50 patients at baseline. Hypertension (56%), hyperlipidaemia (48%), gastro-oesophageal reflux disease (40%), ischaemic heart disease (38%), depression (30%), asthma (28%), diabetes (28%), osteoarthritis (24%), osteoporosis (24%) and anxiety (22%) were the ten most recurring co-morbidities.

**Table 1:** Baseline characteristics of patient participants.

Characteristic	*N* = 50
Sex	Male: 25 (50%)
	Female: 25 (50%)
Age—Mean years (SD)	69.22 (±8.43)
Ethnicity	Caucasian 44 (88%)
	Asian 3 (6%)
	Arabic 2 (4%)
	Other 1 (2%)
Level of education	University 16 (32%)
	Some high school 15 (30%)
	HSC 15 (30%)
	TAFE 3 (6%)
	Primary school 1 (2%)
Employment status	Retired 38 (76%)
	Employed 7 (14%)
	Unemployed 4 (8%)
	Carer 1 (2%)
Living arrangement	With someone 35 (70%)
	Alone 15 (30%)
BMI (SD)	28.99 (±6.19)
Smoking status	Ex-smoker 31 (62%)
	Current smoker 12 (24%)
	Never smoked 7 (14%)
Prior experience of pulmonary rehabilitation	Yes 8 (16%)
	No 42 (84%)
Outcome measures	Mean (SD)
PAM 13	57.68 (±10.83)
COPD-Q	7.24 (±2.12)
CAT	19.22 (±6.95)
MULTIPleS	25.3 (±14.07)
COPD-MULTIPleS	11.76 (±6.64)
Correct inhaler device technique (*n* = 36)	6 (16.67%)

### Change in the study’s outcome measures

As seen in Table 2, there was a significant increase (<0.001) of 7.16 points in the ﻿Patient Activation Measure 13 (PAM 13), the primary outcome measure. There was also a significant increase (<0.001) of 1.75 in the patients’ knowledge of COPD, measured by the COPD Knowledge Questionnaire (COPD-Q), at 6 months’ follow-up. The patients’ COPD-related quality of life was also significantly improved (0.012), with a change of 2.45 points in their COPD Assessment Test (CAT) score.

**Table 2:** Paired comparison of outcome measures of participants who completed 6 months’ follow-up.

Outcome measure	Baseline (SD)	Post-programme (SD)	Significance* (two-tailed)
PAM 13^a^ (*n* = 44)	57.69 (±11.01)	64.85 (±16.22)	<0.001
CAT^a^ (*n* = 42)	20 (±7)	17.55 (±7.7)	0.012
COPD-Q^a^ (*n* = 44)	7.27 (±2.12)	9.02 (±2.14)	<0.001
MULTIPleS^a^ (*n* = 43)	25.58 (±14.15)	26.02 (±11.43)	0.822
COPD-MULTIPleS^a^ (*n* = 42)	12.48 (±6.8)	10.71 (±6.05)	0.084
Proper inhaler device technique^b^ (*n* = 30)	5 (16.67%)	20 (66.67%)	0.001

*Statistical significance was defined as *p* < 0.05 prior to analysis.

^a^Paired *t* test was used to compare the difference in pre- and post-programme outcomes for the five questionnaires with continuous variables.

^b^Pre- and post-programme difference in inhaler device technique, which is a dichotomous variable, was compared using McNemar’s Test.

The change of 0.44 in patients’ perception of the burden of their multi-morbidity, measured by the Multimorbidity Illness Perceptions Scale (MULTIPleS), was not significant (*p* = 0.822), nor was the difference of 1.76 in perception of their COPD in the context of multi-morbidity (*p* = 0.084). There was significant improvement in inhaler device technique (*p* = 0.001), with 50% more patients having proper inhaler technique at 6 months’ follow-up.

Table 3 details the change in outcome measures for a subset of 35 patients who attended all three sessions of the programme. They also had a significant improvement in activation (*p* < 0.001), COPD knowledge (*p* < 0.001), COPD-related quality of life (0.024) and inhaler device technique (0.002). There was no significant change in their perception of multi-morbidity (0.907) or COPD-related multi-morbidity (0.237).

**Table 3:** Paired comparison of outcome measures of subset who completed the programme.

Outcome measure	Baseline (SD)	Post-programme (SD)	Significance* (two-tailed)
PAM 13^a^ (*n* = 35)	59.03 (±10.7)	68.3 (±15.81)	<0.001
CAT^a^ (*n* = 34)	19.47 (±6.42)	16.85 (±7.29)	0.024
COPD-Q^a^ (*n* = 35)	7.6 (±2.14)	9.34 (±1.73)	<0.001
MULTIPleS^a^ (*n* = 35)	25.29 (±13.46)	25.06 (±11.81)	0.907
COPD-MULTIPleS^a^ (*n* = 34)	12.41 (±6.17)	11.09 (±6.4)	0.237
Proper inhaler device technique^b^ (*n* = 28)	5 (17.86%)	18 (64.28%)	0.002

*Statistical significance was defined as *p* < 0.05 prior to analysis.

^a^Paired *t* test was used to compare the difference in pre- and post-programme outcomes for the five questionnaires with continuous variables.

^b^Pre- and post-programme difference in inhaler device technique, which is a dichotomous variable, was compared using McNemar’s Test.

## Discussion

Our findings indicated that the self-management education programme led to significant improvement in patient activation, COPD knowledge, COPD-related quality of life and inhaler device technique. These changes supported the three hypotheses postulated by the study and also support the idea that a theory-based, tailored self-management programme that recognises and responds to the presence of existing co-morbidities may be more effective than current approaches.

There was a statistically significant increase in the patients’ level of activation in terms of their overall health behaviour, as measured by the PAM 13^22^. Even though the mean post-test score of the PAM remained in Stage 3 at 6 months’ follow-up, the increase of 7.16 points was meaningful as a minimum increase of four points following an intervention suggests a clinical improvement in self-efficacy^23^. This increase in the PAM indicated that, on an average, the patients were in control of their own health-related behaviour but needed motivation to adopt and maintain new behaviours^24^.

Prior interventional studies for chronic disease have had similar outcomes in terms of patient activation. A home-based educational intervention for native Americans with type 2 diabetes by Shah et al.^25^ found a significant increase in patient activation. Another self-management intervention for diabetic patients in Norwegian primary care demonstrated a persistent increase in their level of activation^26^. In their secondary analysis of a randomised trial of self-care for patients with depression and co-morbidities, McCusker et al.^27^ found that activation significantly improved in both coached and non-coached groups at 6 months’ follow-up. Another self-management intervention for COPD in the UK by Turner et al.^23^ demonstrated a significant improvement in paired measures of patient activation.

There was significantly improved COPD-related quality of life following the APCOM programme, as assessed by the CAT^28^. This is in contrast to a previous general practice-based study^29^, and to the findings of the systematic review by Jolly et al.^17^, which found no improvement in health-related quality of life as assessed by the St George’s Respiratory Questionnaire. In a trial of self-management for COPD by Bischoff et al.^30^, comprehensive, tailored PN-delivered education and routine monitoring did not show better quality of life or self-efficacy. This could be attributed to the single disease-focussed nature of their intervention. Some prior self-management interventions for COPD have yielded positive outcomes. Another PN-led self-management intervention for patients with COPD in the UK showed a significant improvement in their CAT scores^31^, which are in line with our study’s findings. A pilot trial of self-management support for primary care patients with COPD in the UK saw an improvement in their health-related quality of life at 6 months’ follow-up, but this study only recruited patients with moderate-to-severe disease^14^.

There was a significant improvement in the patients’ knowledge of their COPD following the APCOM programme, which was measured by the COPD-Q^32^. Increase in patients’ COPD knowledge was also observed following an interdisciplinary programme to enhance management of asthma and COPD in primary care^33^. There was no significant change in the patients’ perception of their multi-morbidity, or COPD in the context of multi-morbidity, following the programme. This might be because the patients were already aware of the burden imposed by their various chronic conditions. A British survey that explored factors predicting self-management behaviour^34^ found that patients’ experience of multi-morbidity, based on the MULTIPleS^35^ scale, was not a critical predictor of self-management; self-management behaviour was predicted by illness perceptions around the consequences of individual conditions.

The patients’ inhaler device technique^36^ significantly improved following the programme. It is interesting that no prior self-management interventions for COPD in primary care seemed to have assessed inhaler technique. This might be due to the lack of a standard method for assessing inhaler technique for different types of devices, which imposes a barrier towards its accurate assessment^37^.

To the best of our knowledge, the innovative APCOM programme was a first to provide tailored, self-management support for primary care patients with COPD in the context of multi-morbidity. Unlike other self-management programmes for COPD that used existing interventions and mainly focussed on physical outcomes^30,38^, our multi-faceted programme was newly developed and incorporated a biopsychosocial perspective. This entailed looking at biomedical as well as psychological aspects^39^, and being considerate of patients’ preferences, needs and values, which are essential for providing self-management support for chronic disease^40^. As opposed to other self-management interventions for COPD in primary care which had a single disease focus^13,14^, the APCOM programme considered the implications of patients’ co-morbidities towards their COPD. Patients were included irrespective of the severity of their COPD, unlike a British practice nurse-led, self-management trial of telephone health coaching, which only included patients with mild symptoms of the disease^41^.

All participating general practices and PNs remained in the study, with 12% of the patients being lost to follow-up, which was lower than the anticipated dropout rate of 20%^42^. This shows feasibility of recruitment and retention of practices for a large-scale trial. The response rate of 88% at 6 months’ follow-up in our study was higher than a group self-management initiative for COPD, which also used PAM 13 as the primary outcome measure but had a response rate of 47%^23^. This is further indication of the effectiveness of personalised self-management support for empowering patients with COPD. The sample size of our study was adequate for test−retest reliability of the outcome measures^43^. Although a confidence interval of about 80% is deemed sufficient for analysis of data from pilot studies^44^, the confidence interval for analysis of the study’s endpoints was set to 95%.

A major limitation of our study was the lack of a control group, which makes it difficult to ascertain whether post-programme improvement in the outcome measures were solely due to the programme^43^. Inclusion of a control group would have required 30 more patient participants (*N* = 80), with 40 in each group to estimate test−retest reliability^42^. This would have required at least six more PNs and practices, further extending the time for practice recruitment, which was about 8 months for our study. Given that the main purpose of this study was to pilot test the effect of a complex intervention in a real-world clinical setting, it was not essential to include a control group^45^.

Given the positive finding from our pilot study, a large-scale randomised controlled trial based on findings of the APCOM study is recommended as the next step for research. Such a study needs to be adequately powered to show a difference in health-related quality of life and health service utilisation. Interventional studies are needed for Indigenous patients with COPD and co-morbidities, in view of greater incidence of the disease in Indigenous Australians. Initiatives to deliver such tailored, self-management support for other chronic conditions need to be implemented in routine general practice, irrespective of who delivers the programme. Patients should be educated regarding the correct inhaler device technique upon initial prescription, and their technique should be assessed regularly during GP and PN consultations.

In conclusion, the tailored, self-management education programme piloted in our study led to improvement in patients’ self-efficacy in terms of their COPD and overall health behaviour. There was significant increase in patient activation, COPD knowledge, COPD-related quality of life and inhaler device technique at 6 months’ follow-up. There was no significant difference in patients’ perception of their multi-morbidity or COPD-related multi-morbidity. The study echoes the growing need for personalised self-management support for COPD in the context of multi-morbidity at the primary care level. The findings are an empirical basis for testing the innovative programme as a future large-scale randomised controlled trial.

## Methods

The APCOM study was a single-group, pre- and post-intervention study conducted in participating general practices across metropolitan Sydney from mid-2015 to December 2016. The study comprised development, piloting and evaluation of an innovative self-management education programme for COPD in the context of multi-morbidity. The study protocol and components of the programme have been described in detail elsewhere^46^. Ethics approval was obtained from the Human Research Ethics Committee of UNSW Sydney (HREC14139).

### Participant eligibility

General practices were eligible to participate if they maintained an electronic database of medical records and employed one or more PN. Patients were included if they: (i) were aged between 40 and 84 years, (ii) had a record of spirometry diagnosis of COPD in their practice notes and (iii) had at least one other co-existing chronic condition or co-morbidity. Patients were excluded if they had cognitive impairment or were unable to understand sufficient English to complete the study questionnaires and follow the intervention.

### Recruitment

General practices were invited to participate from the Practice Based Research Network at UNSW Sydney, a group of practices that had previously participated in research studies conducted by N.Z. and S.D., or expressed an interest in research participation. An information sheet about the study was faxed to potential general practices. Practices that did not respond within 2 weeks of the initial invitation were followed up twice by telephone and/or email. S.A. visited practices that expressed an interest in taking part in the study and met with the PNs and GPs, briefed them about the study and obtained their written informed consent.

The PNs were provided with instructions on how to search for eligible patients in the practice records based on the inclusion criteria. All potentially eligible patients were sent an invitation letter, including a brief description of the study. Non-responders were reminded by the PNs via telephone after 2 weeks. Each PN was asked to recruit five patients for the study since this number seemed feasible to all.

### Intervention

The details of the intervention have been included in the published study protocol^46^. In brief, participating PNs attended 1-day workshops facilitated by the authors and were trained to deliver the self-management education programme. The PNs were trained to conduct a patient assessment using a template based on the Health Belief Model^47^. This assessment identified the patients’ health priorities and was used by the PNs to facilitate development of tailored strategies for the patients to support self-management of COPD in the context of multi-morbidity. One of the authors (S.A.) provided research support to the PNs during the 6-week programme, which comprised three face-to-face sessions spaced 2 weeks apart.

During the first PN−patient session, individual patient needs were assessed and the intervention tailored accordingly. The PNs were asked to use Motivational Interviewing^48^ to address barriers faced by the patients in managing their COPD in the face of co-morbidities. Health information and referrals to healthcare providers were provided as necessary. Following the last session, the PNs followed up with the patients via a monthly phone call for 5 months.

### Outcome measures

The primary outcome was the Patient Activation Measure (PAM 13)^22^. Secondary outcomes were COPD Assessment Test (CAT)^28^, COPD Knowledge Questionnaire (COPD-Q)^32^, Multimorbidity Illness Perceptions Scale (MULTIPleS)^35^, COPD-specific version of MULTIPleS and inhaler device technique^36^. The first hypothesis of the study was addressed by the PAM 13, COPD-Q, CAT, COPD-MULTIPleS and inhaler device technique, the second hypothesis was addressed by the COPD-Q, CAT and inhaler device technique, and the third hypothesis was addressed by the PAM 13 and MULTIPleS.

### Reporting summary

Further information on research design is available in the Nature Research Reporting Summary linked to this article.

## Supplementary information

Reporting Summary

## Data Availability

The datasets generated during and/or analysed during the current study are available from the corresponding author on reasonable request.
